# Supplementary material for: The Hatching Time of Broiler Chickens Modifies Not Only the Production Traits but Also the Early Bacteriota Development of the Ceca
Source: Animals (Basel). 2023 Aug 25;13(17):2712. doi: 10.3390/ani13172712 (PMC10487082; doi:10.3390/ani13172712)
Supplement: Supplementary file 1 [file animals-13-02712-s001.zip › animals-2550454-supplementary.pdf]

Supplementary Material

The hatching time of broiler chickens modifies not only the production traits, but also the bacteriota development in the ceca

Nikoletta Such [1], Kornél Schermann [1], László Pál [1], László Menyhárt [2], Valéria Farkas [1], Gábor Csitári [1], Brigitta Kiss [1], Kesete Goitom Tewelde [1] and Károly Dublecz [1]

[1] Institute of Physiology and Nutrition, Hungarian University of Agriculture and Life Sciences, Georgikon Campus, 8360 Keszthely, Hungary; Deák Ferenc Street 16.; such.nikoletta.amanda@uni-mate.hu; schermannkornel2000@gmail.com; pal.laszlo@uni-mate.hu; farkas.valeria@uni-mate.hu; csitari.gabor@uni-mate.hu; kiss.brigitta.gyongyi@phd.uni-mate.hu; kesete.goitom.tewelde@phd.uni-mate.hu; dublecz.karoly@uni-mate.hu

[2] Institute of Technology, Hungarian University of Agriculture and Life Sciences, Georgikon Campus, Deák Ferenc Street 16, 8360 Keszthely, Hungary; menyhart.laszlo@uni-mate.hu

* Correspondence: dublecz.karoly@uni-mate.hu

† These authors contributed equally to this work.

Table S1. The effect of hatching time and parent age on the caecal microbiota composition at the family level at 11 day of age.

Family (%)	Hatching time	Parent flock age			FDR p-values		
		Young parent	Old parent	Average (Hatching time)	Hatching time	Parent flock age	Interaction
Ruminococcaceae	Early	35.051	33.467	34.259	0.443		0.635
	Late	29.792	24.768	27.280			
	Average (Age)	32.422	29.118			0.750	
Lachnospiraceae	Early	22.571	30.636	26.604	0.886		0.846
	Late	27.650	23.050	25.350			
	Average (Age)	25.111	26.843			0.683	
Lactobacillaceae	Early	14.110	12.439	13.275	0.504		0.729
	Late	10.458	7.046	8.752			
	Average (Age)	12.284	9.743			0.717	
Bacteroidaceae	Early	9.662	5.952	7.807	0.517		0.933
	Late	12.424	27.494	19.959			
	Average (Age)	11.043	16.723			0.742	
Erysipelotrichaceae	Early	4.533	5.118	4.826	0.542		0.425
	Late	2.845	3.680	3.262			
	Average (Age)	3.689	4.399			0.702	
Peptostreptococcaceae	Early	0.318	1.073	0.695	0.533		0.921
	Late	0.232	0.372	0.302			
	Average (Age)	0.275	0.722			0.816	
Clostridiales_vadinBB60_group	Early	3.098	5.917	4.508	0.294		0.634
	Late	7.171	7.921	7.546			
	Average (Age)	5.135	6.919			0.849	
Enterococcaceae	Early	0.435	1.172	0.804	0.849		0.707
	Late	0.895	0.518	0.706			
	Average (Age)	0.665	0.845			0.695	
Enterobacteriaceae	Early	0.153	0.420	0.287	0.365		1.447
	Late	1.929	0.398	1.163			
	Average (Age)	1.041	0.409			0.965	
Christensenellaceae	Early	0.192	0.124	0.158	0.589		0.553
	Late	0.103	0.052	0.077			
	Average (Age)	0.148	0.088			0.700	
Defluviitaleaceae	Early	0.108	0.124	0.116	0.959		0.914
	Late	0.099	0.129	0.114			
	Average (Age)	0.103	0.127			0.699	
Eggerthellaceae	Early	0.071	0.088	0.079	0.374		0.950
	Late	0.040	0.063	0.051			
	Average (Age)	0.055	0.075			0.910	
Family_XIII	Early	0.075	0.064	0.070	0.708		0.912
	Late	0.076	0.030	0.053			

	Average (Age)	0.076	0.047			0.832	
Pseudomonadaceae	Early	0.006	0.008	0.007	0.502		0.736
	Late	0.002	0.001	0.002			
	Average (Age)	0.004	0.005			0.916	
Staphylococcaceae	Early	0.003	0.000	0.002	0.990		0.818
	Late	0.004	0.000	0.002			
	Average (Age)	0.004	0.000			1.131	
Anaeroplasmataceae	Early	3.444	1.118	2.281	0.513		0.850
	Late	1.788	0.275	1.032			
	Average (Age)	2.616	0.697			0.526	
Bacillaceae	Early	4.044	1.726	2.885	0.837		0.805
	Late	3.246	3.838	3.542			
	Average (Age)	3.645	2.782			0.664	
Clostridiaceae_1	Early	0.257	0.222	0.239	0.888		0.411
	Late	0.659	0.022	0.340			
	Average (Age)	0.458	0.122			0.669	
Not_Assigned	Early	1.560	0.149	0.854	0.535		0.895
	Late	0.339	0.007	0.173			
	Average (Age)	0.950	0.078			0.805	
Peptococcaceae	Early	0.027	0.012	0.019	0.835		0.692
	Late	0.014	0.035	0.024			
	Average (Age)	0.020	0.023			0.913	
Rikenellaceae	Early	0.001	0.000	0.001	0.720		0.970
	Late	0.000	0.008	0.004			
	Average (Age)	0.001	0.004			0.680	
Streptococcaceae	Early	0.000	0.043	0.021	0.595		1.116
	Late	0.002	0.000	0.001			
	Average (Age)	0.001	0.021			0.680	

Table S2. The effect of hatching time and parent age on the caecal microbiota composition at the family level at 39 day of age.

Family (%)	Hatching time	Parent flock age			FDR p-values		
		Young parent	Old parent	Average (Hatching time)	Hatching time	Parent flock Age	Interaction
Ruminococcaceae	Early	29.016	28.005	28.510	0.911		0.762
	Late	30.406	27.834	29.120			
	Average (Age)	29.711	27.919			0.639	
Lachnospiraceae	Early	25.967	20.575	23.271	0.892		0.680
	Late	23.783	26.714	25.249			
	Average (Age)	24.875	23.645			0.863	
Lactobacillaceae	Early	25.336	32.926	29.131	0.735		0.657
	Late	24.549	20.733	22.641			
	Average (Age)	24.943	26.830			0.861	
Bacteroidaceae	Early	2.984	1.325	2.155	1.012		0.896
	Late	2.628	1.709	2.169			
	Average (Age)	2.806	1.517			1.016	
Erysipelotrichaceae	Early	2.016	1.862	1.939	0.613		0.785
	Late	1.822	3.414	2.618			
	Average (Age)	1.919	2.638			0.979	
Peptostreptococcaceae	Early	0.822	0.857	0.839	1.015		0.810
	Late	1.401	2.214	1.807			
	Average (Age)	1.112	1.535			0.604	
Clostridiales_vadinBB60_group	Early	3.259	2.806	3.032	0.963		0.853
	Late	3.651	2.622	3.136			
	Average (Age)	3.455	2.714			0.720	
Enterococcaceae	Early	0.081	0.482	0.282	0.862		0.789
	Late	0.078	0.257	0.167			
	Average (Age)	0.080	0.370			0.647	
Enterobacteriaceae	Early	0.142	0.113	0.128	0.689		0.824
	Late	0.449	0.335	0.392			
	Average (Age)	0.296	0.224			0.844	
Christensenellaceae	Early	1.529	0.976	1.252	0.847		0.739
	Late	1.256	1.703	1.479			
	Average (Age)	1.392	1.340			0.951	
Defluviitaleaceae	Early	0.185	0.100	0.142	0.938		0.488
	Late	0.117	0.152	0.135			
	Average (Age)	0.151	0.126			0.628	
Eggerthellaceae	Early	0.021	0.029	0.025	0.858		0.814
	Late	0.017	0.042	0.030			
	Average (Age)	0.019	0.035			1.921	
Family_XIII	Early	0.220	0.234	0.227	0.575		0.675
	Late	0.131	0.218	0.174			

	Average (Age)	0.175	0.226			0.639	
Pseudomonadaceae	Early	0.000	0.005	0.002	0.957		0.803
	Late	0.002	0.003	0.003			
	Average (Age)	0.001	0.004			0.588	
Staphylococcaceae	Early	0.004	0.000	0.002	0.860		0.925
	Late	0.012	0.003	0.007			
	Average (Age)	0.008	0.001			0.755	
Anaeroplasmataceae	Early	0.008	0.063	0.036	0.812		0.828
	Late	0.002	0.034	0.018			
	Average (Age)	0.005	0.049			0.844	
Bacillaceae	Early	1.465	2.362	1.914	0.884		0.647
	Late	1.671	0.717	1.194			
	Average (Age)	1.568	1.540			0.966	
Clostridiaceae_1	Early	0.000	0.007	0.003	0.953		0.842
	Late	0.033	0.216	0.124			
	Average (Age)	0.016	0.112			0.605	
Not_Assigned	Early	0.849	0.962	0.906	0.878		0.762
	Late	1.136	0.949	1.042			
	Average (Age)	0.992	0.955			0.965	
Peptococcaceae	Early	0.514	0.423	0.469	0.880		0.767
	Late	0.594	0.425	0.510			
	Average (Age)	0.554	0.424			0.742	
Rikenellaceae	Early	0.018	0.003	0.010	0.814		0.650
	Late	0.003	0.007	0.005			
	Average (Age)	0.011	0.005			0.576	
Streptococcaceae	Early	0.426	0.044	0.235	0.747		0.695
	Late	0.589	1.844	1.217			
	Average (Age)	0.508	0.944			0.622	
